# Microtubule associated protein 4 (MAP4) phosphorylation reduces cardiac microvascular density through NLRP3-related pyroptosis

**DOI:** 10.1038/s41420-021-00606-w

**Published:** 2021-08-12

**Authors:** Yan-hai Feng, Ling-fei Li, Qiong Zhang, Jun-hui Zhang, Yao Huang, Yan-ling Lv, Jie-Zhi Jia, Dongxia Zhang, Jiong-Yu Hu, Yue-Sheng Huang

**Affiliations:** 1 Laboratory Institute of Burn Research, Southwest Hospital, Third Military Medical University (Army Medical University), Chongqing, China; 2 State Key Laboratory of Trauma, Burns and Combined Injury, Southwest Hospital, Third Military Medical University (Army Medical University), Chongqing, China; 3 Department of Dermatology, Daping Hospital, Third Military Medical University (Army Medical University), Chongqing, China; 4 Endocrinology Department, Southwest Hospital, Third Military Medical University (Army Medical University), Chongqing, China; 5 Department of Wound Repair, Institute of Wound Repair, Shenzhen People’s Hospital, First Affiliated Hospital of Southern University of Science and Technology, Shenzhen, China

**Keywords:** Mechanisms of disease, Cardiac hypertrophy

## Abstract

Phosphorylation of MAP4 (p-MAP4) causes cardiac remodeling, with the cardiac microvascular endothelium being considered a vital mediator of this process. In the current study, we investigated the mechanism underlying p-MAP4 influences on cardiac microvascular density. We firstly confirmed elevated MAP4 phosphorylation in the myocardium of MAP4 knock-in (KI) mice. When compared with the corresponding control group, we detected the decreased expression of CD31, CD34, VEGFA, VEGFR2, ANG2, and TIE2 in the myocardium of MAP4 KI mice, accompanied by a reduced plasma concentration of VEGF. Moreover, we observed apoptosis and mitochondrial disruption in the cardiac microvascular endothelium of MAP4 KI animals. Consistently, we noted a decreased cardiac microvascular density, measured by CD31 and lectin staining, in MAP4 KI mice. To explore the underlying mechanism, we targeted the NLRP3-related pyroptosis and found increased expression of the corresponding proteins, including NLRP3, ASC, mature IL-1β, IL-18, and GSDMD-N in the myocardium of MAP4 KI mice. Furthermore, we utilized a MAP4 (Glu) adenovirus to mimic cellular p-MAP4. After incubating HUVECs with MAP4 (Glu) adenovirus, the angiogenic ability was inhibited, and NLRP3-related pyroptosis were significantly activated. Moreover, both cytotoxicity and PI signal were upregulated by the MAP4 (Glu) adenovirus. Finally, NLRP3 inflammasome blockage alleviated the inhibited angiogenic ability induced by MAP4 (Glu) adenovirus. These results demonstrated that p-MAP4 reduced cardiac microvascular density by activating NLRP3-related pyroptosis in both young and aged mice. We thus managed to provide clues explaining MAP4 phosphorylation-induced cardiac remodeling and enriched current knowledge regarding the role of MAP4.

## Introduction

Cardiac remodeling, characterized by cardiac fibrosis and hypertrophy, is a cellular response to abundant stimuli. It is often attributed to sustained pathological stimuli, including hypoxia and myocardial infarction (MI) [1]. Pathological cardiac remodeling is a major cause for the development of diastolic and systolic dysfunction, and ultimately heart failure, resulting in high mortality (~50% at 5 y) [2, 3]. Improvement of cardiac remodeling has been reported to delay or even prevent the progression of heart failure. The vascular endothelium is an essential constituent of the heart, and endothelial cell homeostasis is known to sustain normal physiological cardiac functions. In contrast, the endothelium dysfunction reportedly influences pathological cardiac remodeling, especially after myocardial infarction [4]. We have previously demonstrated that p-MAP4 caused cardiac remodeling in an age-dependent manner [1] and observed decreased microvascular densities in the myocardium of MAP4 knock-in (KI) mice in further studies. Therefore, a more comprehensive understanding of the molecular mechanisms (especially from the aspect of endothelial cells) of p-MAP4-induced pathological cardiac remodeling might encourage the establishment of novel therapeutic approaches to manage heart failure.

Microtubule-associated proteins (MAPs) are well-known cytosolic skeleton proteins that promote microtubule (MT) polymerization under physiological conditions. These assembly-promoting MAPs include MAP2, tau, and MAP4. As one of the most intensively studied, MAP4 is ubiquitously expressed in nonneural cells, and its function is mainly regulated through its phosphorylation [1, 5, 6]. As reported, the S696, S768, and S787 sites in the proline-rich region of the MT-binding domain of human MAP4 are pivotal sites for phosphorylation, modulating its separation from MTs [1, 7, 8]. Furthermore, MT destabilization is considered a core impactor of endothelial function [9, 10]. Endothelial cells have close contact with various circulating factors in the blood, which temporally and spatially regulate the homeostasis of the vascular microenvironment. Our group has reported that p-MAP4 promoted the migration and proliferation of endothelial cells but disrupted the endothelial barrier function, one of the most critical biological behaviors of endothelial cells [5, 11]. Therefore, cardiac microvascular intensity, the end stage of angiogenesis, another major function of endothelial cells, might also be influenced by p-MAP4. As such, its investigation should be of significance.

Inflammatory responses play a critical role in determining the extent of cardiac remodeling. As a newly discovered inflammation-related programmed cell death process, pyroptosis has been reported to occur in multiple organs or cells, including heart and endothelial cells, under several conditions [12, 13]. Inflammasomes, essential components of pyroptosis and the principal initiator of the innate immune response, can be divided into various categories. One of the most familiar, the NLRP3 inflammasome, consists of NLRP3 (NLR family pyrin domain containing 3), PYCARD or ASC (apoptosis-associated speck-like protein containing a CARD), and caspase-1. Along with the activation of NLRP3 inflammasome, pro-caspase-1 is catalyzed into its active form, further cleaving gasdermin (GSDM) family proteins, leading to pyroptosis [14]. Among the GSDM family, gasdermin D (GSDMD) is regarded as the primary effector molecule and is constitutively autoinhibited by the binding of its C-terminal (GSDMD-C) repressor domain to its N-terminal (GSDMD-N) pore-forming domain [15]. During the induction of pyroptosis, inflammatory caspases, mainly caspase-1, have been reported to ease this autoinhibition by catalyzing the proteolytic cleavage of the interdomain loop, causing the liberation of the GSDMD-N pyroptotic inducer [16, 17]. The cleaved GSDMD-N then translocates to the inner leaflet of the plasma membrane, with membrane targeting enabling GSDMD-N oligomerization, producing a pore that stimulates rapid plasma membrane permeabilization, resulting in cell swelling and death [15]. In addition, p-MAP4 is activated by inflammatory molecules, including tumor necrosis factor α (TNF-α) and lipopolysaccharides (LPS) [11]. In particular, LPS served as a conventional inducer of the NLRP3 inflammasome in various models [18]. Therefore, there is a connection between p-MAP4 and NLRP3-related pyroptosis, warranting further studies.

Here, we investigated the effects of p-MAP4 on cardiac microvascular density and the roles of NLRP3-related pyroptosis during this process. We found that p-MAP4 reduced cardiac microvascular density in both young and aged MAP4 KI mice. The molecular mechanism was shown to be associated with NLRP3-related pyroptosis. Thus, MAP4 might play a significant role in maintaining cardiac microvascular density, providing a new potential therapeutic strategy for cardiac remodeling.

## Results

### Inhibited angiogenic signaling pathways in the myocardium of MAP4 KI mice

**Fig. 1:**
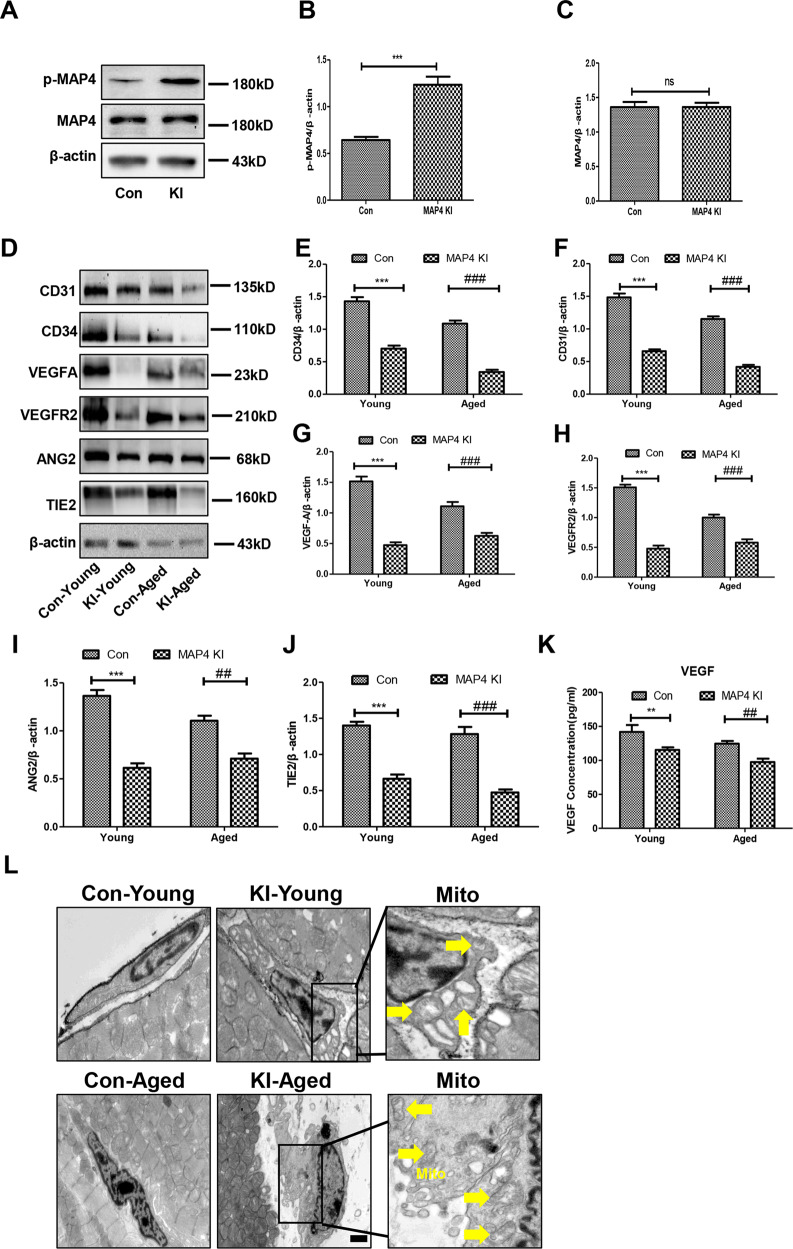
Angiogenic signaling pathways are inhibited in the myocardium of MAP4 KI mice. **A** Representative bands of western blotting for detecting p-MAP4 in the myocardium; *n* = 5. **B**, **C** The statistical analysis of p-MAP4, and MAP4. ***p < 0.001 versus the Con group. N.S. represents no statistical difference between the Con and MAP4 KI groups; *n* = 5. **D** Representative immunoblotting bands of the levels of CD31, CD34, VEGFA, VEGFR2, ANG2, and TIE2 in the myocardium; *n* = 5. **E**–**J** The statistical analysis of CD31, CD34, VEGFA, VEGFR2, ANG2, and TIE2. ***p < 0.001 versus the Con-young group; ^##^*p* < 0.01 and ^###^*p* < 0.001 versus the Con-aged group; *n* = 5. **K** A scatter diagram showing the concentration of VEGF in the plasma. N.S. represents no statistical difference between the Con-young and MAP4 KI-young groups. ^##^*p* < 0.01 versus the Con-aged group; *n* = 8. **L** Representative TEM images of endothelial cells in the myocardium. Yellow arrows point to disrupted mitochondria. Scale bar, 0.5 µm; *n* = 3.

The contribution of p-MAP4 to cardiac remodeling, especially in aged mice, was recently revealed [1]. In a recent study, we investigated the cardiac vascular microenvironment which was an essential mediator of cardiac remodeling [19]. To study the microvascular state in the myocardium of the MAP4 KI mouse, we first aimed to confirm whether this mouse could be used as a MAP4 phosphorylation model. As shown in Fig. 1A, the level of p-MAP4 was increased (Fig. 1A–C, *p* < 0.05). Thereby validating the MAP4 KI mouse as an effective p-MAP4 model.

Next, we analyzed the markers of endothelial cell and angiogenic signaling pathways. All aged mice, regardless of being control or MAP4 KI mice, were assured according to our previous research [1]. Accordingly, as shown in Fig. 1B, the expression of CD31 and CD34 in the myocardium of MAP4 KI mice was reduced in both young and aged mice compared with the corresponding control group (Fig. 1D–F, *p* < 0.05). Meanwhile, the levels of VEGFA, VEGFR2, ANG2, and TIE2, the principal signaling pathways of angiogenesis, exhibited a similar variation tendency as that of CD31 (Fig. 1D, G–J, *p* < 0.05). To further verify the concentration of VEGF in plasma, we performed ELISA. Our results showed that VEGF expression was decreased in MAP4 KI mice compared with the control group, in both young and aged mice (Fig. 1K, *p* < 0.001). Besides, we visualized the morphology of endothelial cells in the myocardium using TEM. Furthermore, we observed the disrupted mitochondria in the cardiac endothelial cells of both young and aged MAP4 KI mice when compared with the corresponding control group (Fig. 1L). These data indicated that the two angiogenic signaling pathways were inhibited, and the mitochondria of endothelial cells was disrupted in MAP4 KI mice.

### Reduced cardiac microvascular density in MAP4 KI mice

**Fig. 2:**
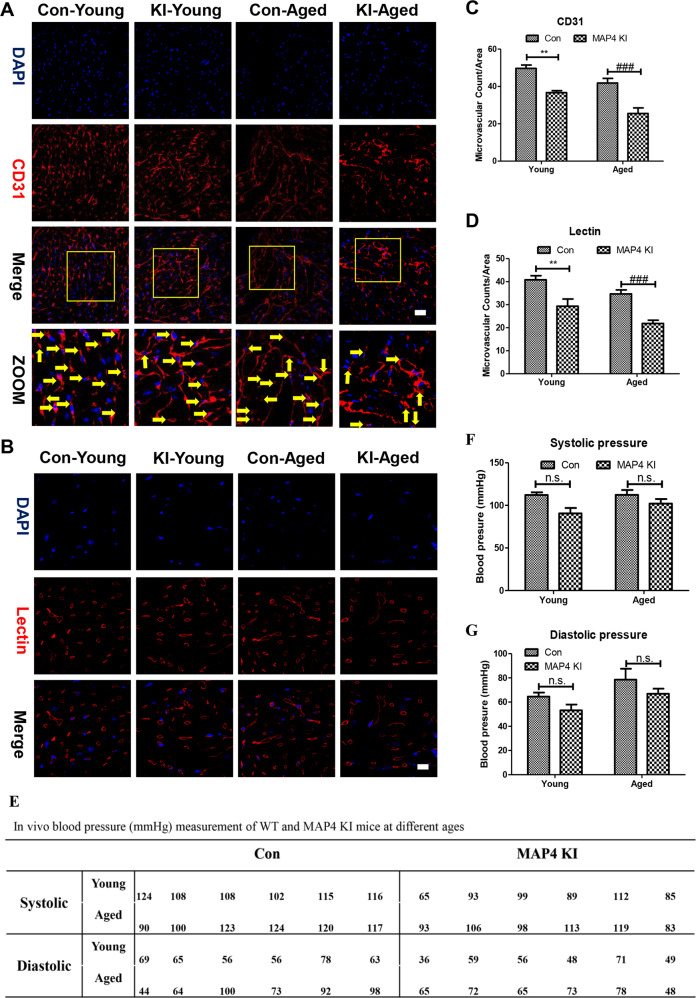
Reduced cardiac microvascular density in the myocardium of MAP4 KI mice. **A** Representative confocal images of CD31 in the myocardium. Scale bar, 10 µm; *n* = 3. Yellow arrows point to capillary. **B** Representative confocal images of lectin in the myocardium. Scale bar, 10 µm; *n* = 3. **C** A scatter diagram showing the statistical analysis of microvascular density in myocardium stained with CD31. N.S. represents no statistical difference between the Con-young and MAP4 KI-young groups. **p < 0.01 versus the Con-young group; ^###^*p* < 0.001 versus the Con-aged group. **D** A scatter diagram showing the statistical analysis of red circles in myocardium stained with lectin. **p < 0.01 versus the Con-young group; ^###^*p* < 0.001 versus the Con-aged group. **E** In vivo blood pressure measurement of WT and MAP4 KI mice at different ages. **F** Statistical analysis of Systolic pressure in (**A**). N.S. represents no statistical difference; *n* = 6. **G** Statistical analysis of Diastolic pressure in (**A**). N.S. represents no statistical difference; *n* = 6.

Based on the previously mentioned results, we considered it valuable to directly measure the microvascular density using immunofluorescence. Therefore, we first stained for CD31, a marker of both endothelial cells and the microvasculature. As shown in Fig. 2A and C, we observed a reduced quantity of small dots in the CD31-stained myocardium of MAP4 KI mice, in both young and aged MAP4 KI mice (*p* < 0.01).

Furthermore, we detected lectin, another essential indicator of the microvasculature. As shown in Fig. 2B and D, the quantity of stained cardiac microvasculature, as well as the fluorescence intensity of lectin, were visibly decreased in both young and aged MAP4 KI mice when compared with corresponding control mice. Furthermore, we noted a striking difference in both young and aged MAP4 KI mice (Fig. 2D, *p* < 0.01).

Moreover, to identify whether p-MAP4 also impacted peripheral great vessels, we measured the in vivo blood pressure of WT and MAP4 KI mice at different ages. As shown in Fig. 2E–G, at every age group, both systolic and diastolic pressure showed no significant difference between WT and MAP4 KI mice (*p* > 0.05).

Taking together, these results showed that p-MAP4 had no influence on peripheral great vessels, but reduced the cardiac microvascular density of young and aged mice.

### Activated NLRP3-related pyroptosis in the myocardium of MAP4 KI mice

**Fig. 3:**
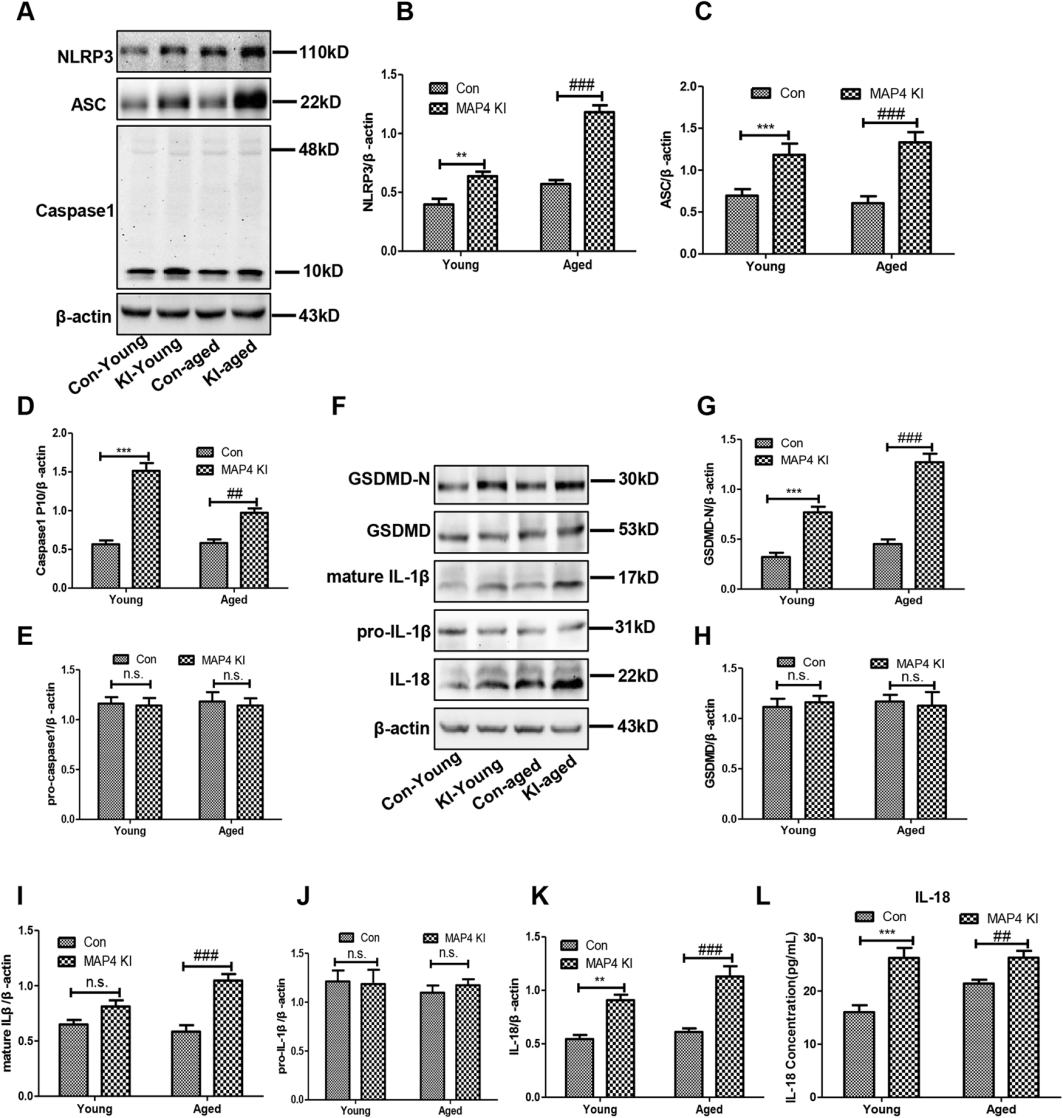
Activated NLRP3-related pyroptosis in the myocardium of MAP4 KI mice. **A** Representative immunoblotting bands of NLRP3, ASC, caspase-1 (p10), and pro-caspase-1 in the myocardium; *n* = 5. **B**–**E** The statistical analysis of NLRP3, ASC, and Caspase-1. **p < 0.01 and ***p < 0.001 versus the Con-young group. ^##^*p* < 0.01 and ^###^*p* < 0.001 versus the Con-aged group. N.S. represents no statistical difference; *n* = 5. **F** Representative immunoblotting bands of GSDMD-N, GSDMD, pro-IL-1β, mature IL-1β, and IL-18 in the myocardium; *n* = 5. **G**–**K** The statistical analysis of GSDMD-N, GSDMD, pro-IL-1β, mature IL-1β, and IL-18. **p < 0.01 and ***p < 0.001 versus the Con-young group. ^###^*p* < 0.001 versus the Con-aged group. N.S. represents no statistical difference; *n* = 5. **L** A scatter diagram showing the concentration of IL-18 in the plasma. ***p < 0.001 versus the Con-young group. ^##^*p* < 0.001 versus the Con-aged group; *n* = 8.

The NLRP3 inflammasome has been reported as a therapeutic target in cardiovascular diseases [20]. Therefore, to determine the possible explanation for these variations mentioned previously, we aimed to evaluate the role of the NLRP3 inflammasome. As shown in Fig. 3A–E, the NLRP3 inflammasome-related proteins, including NLRP3, ASC, and caspase-1 (p10), dramatically increased in the myocardium of MAP4 KI mice when compared with corresponding control groups. These data demonstrated that the NLRP3 inflammasome was activated in the myocardium of MAP4 KI mice, both in young and aged MAP4 KI mice.

We further examined the involvement of pyroptosis, which is closely associated with NLRP3 inflammasome, in cardiovascular diseases [21]. It is known that GSDMD, especially its N-terminal (GSDMD-N), is the core effector molecule in pyroptosis owing to its pore-forming activity [14, 17]. Following the formation of membrane pores, inflammatory cytokines (IL-1β and IL-18) are successively secreted. Therefore, measurement of these molecules allows identifying the condition of pyroptosis in the myocardium of MAP4 KI mice. As shown in Fig. 3F–K, we observed upregulated levels of GSDMD-N and mature IL-1β in the myocardium of MAP4 KI mice (*p* < 0.05). In addition, we further detected the concentration of IL-18 in the plasma using ELISA and observed an augmented IL-18 content in the plasma of MAP4 KI mice, in both young and aged MAP4 KI mice (Fig. 3L, *p* < 0.001). These results indicated that pyroptosis in the myocardium was triggered by p-MAP4 in vivo.

### Inhibited angiogenesis and activated NLRP3 inflammasome were induced by p-MAP4 in vitro

**Fig. 4:**
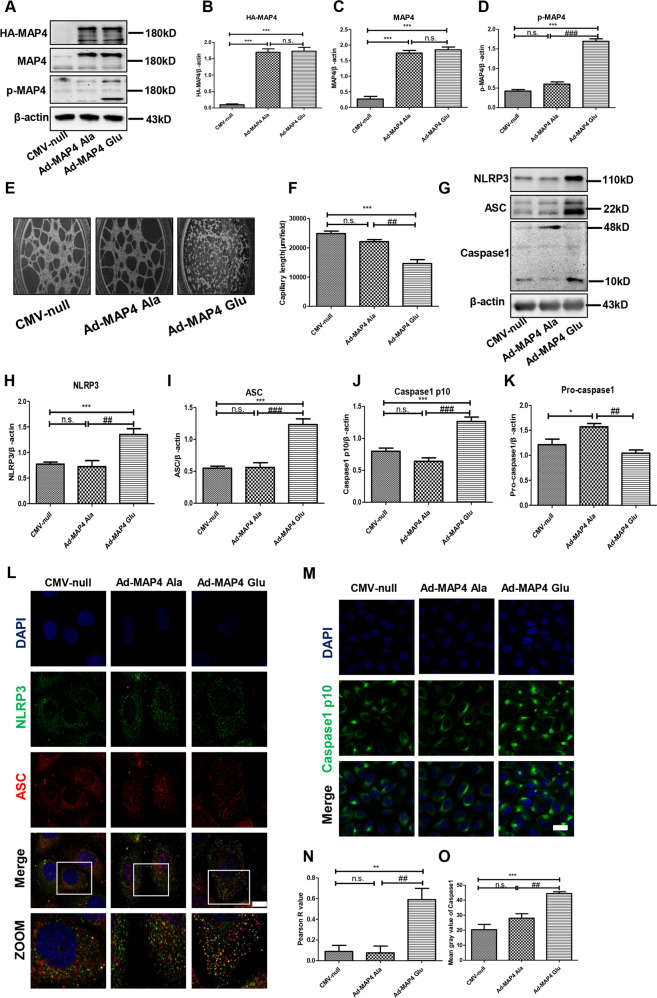
Inhibited angiogenesis and activated NLRP3 inflammasome was induced by p-MAP4 in vitro. **A** Infection efficiency of MAP4 (Glu) or MAP4 (Ala) adenoviruses determined by western blotting; *n* = 5. **B**–**D** The statistical analysis of HA-MAP4, MAP4, and p-MAP4. ***p < 0.001 versus the CMV-null group. ^###^*p* < 0.001 versus the Ad-MAP4 (Ala) group. N.S. represents no statistical difference; *n* = 5. **E** Representative images of tube formation assay; *n* = 3. **F** Capillary length analysis of tube formation assay. ***p < 0.001 versus the CMV-null group. ^##^*p* < 0.001 versus the Ad-MAP4 (Ala) group. N.S. represents no statistical difference; *n* = 3. **G** Representative immunoblotting bands of NLRP3, ASC, caspase-1 (p10), and pro-caspase-1 in HUVECs; *n* = 5. **H**–**K** The statistical analysis of NLRP3, ASC, and caspase-1. *p < 0.05 and ***p < 0.001 versus the CMV-null group. ^##^*p* < 0.01 and ^###^*p* < 0.001 versus the Ad-MAP4 (Ala) group. N.S. represents no statistical difference; *n* = 5. **L** Representative confocal images of NLRP3/ASC colocalization in HUVECs. Scale bar, 10 µm; *n* = 3. **M** Representative confocal images of caspase-1 p10 in HUVECs. Scale bar, 10 µm; *n* = 3. **N** Statistical analysis of Pearson R value of NLRP3/ASC colocalization in HUVECs. **p < 0.01 versus the CMV-null group. ^##^*p* < 0.01 versus the Ad-MAP4 (Ala) group. N.S. represents no statistical difference; n = 3. (**O**) Statistical analysis of mean gray value of caspase-1 p10 in HUVECs. ***p < 0.001 versus the CMV-null group. ^##^*p* < 0.01 versus the Ad-MAP4 (Ala) group. N.S. represents no statistical difference; *n* = 3.

Furthermore, to evaluate the effect of p-MAP4 on the NLRP3 inflammasome in endothelial cells, we used MAP4 (Glu) and MAP4 (Ala) adenoviruses to simulate the phosphorylation or dephosphorylation of MAP4 in vitro, respectively. Considering that the corresponding CMV-null of the two adenoviruses has been consistently shown no effects on the phosphorylation or dephosphorylation of MAP4, we included it for verification in the current study [1, 5, 6, 11, 22]. In addition, we confirmed the roles of MAP4 (Glu) and MAP4 (Ala) on the phosphorylation of MAP4. As shown in Fig. 4A, following the incubation of cells with MAP4 (Glu) or MAP4 (Ala) adenoviruses for 48 h, the levels of HA-MAP4 and MAP4 were significantly elevated compared with the CMV-null-treated group (Fig. 4B and C, *p* < 0.05). Meanwhile, MAP4 (Glu) was shown to successfully induce the intracellular phosphorylation of MAP4 (Fig. 4D, *p* < 0.05), whereas the MAP4 (Ala) adenovirus showed no effects on p-MAP4. These results indicated that the MAP4 (Glu) adenovirus effectively mimicked the intracellular phosphorylation of MAP4 in vitro.

Next, we conducted a tube formation assay as a direct way to determine the influence of p-MAP4 on angiogenesis. As shown in Fig. 4E, the angiogenic abilities of endothelial cells were significantly inhibited by p-MAP4. Moreover, the capillary length of the tube in the Ad-MAP4 (Glu) group was significantly shorter than that of the CMV-null and Ad-MAP4 (Ala) groups (Fig. 4F, *p* < 0.01). These results indicated that p-MAP4 inhibited angiogenesis in vitro.

Next, we analyzed the NLRP3 inflammasome in MAP4 (Glu) adenovirus-treated HUVECs. As shown in Fig. 4G, compared with the CMV-null group, the NLRP3 inflammasome-related proteins, including NLRP3, ASC, and caspase-1 (p10), was upregulated by the MAP4 (Glu) adenovirus, but not by the MAP4 (Ala) adenovirus (Fig. 4H–K, *p* < 0.05). In addition, we employed immunofluorescence to further clarify the NLRP3/ASC colocalization and caspase-1 (p10) generation. As shown in Fig. 4L, when compared with CMV-null and Ad-MAP4 (Ala) group, the NLRP3/ASC colocalization was markedly increased in Ad-MAP4 (Glu) group. In line with this, a higher Pearson R value was observed in Ad-MAP4 (Glu) group (Fig. 4N, *p* < 0.01). Meanwhile, the fluorescence intensities of caspase-1(p10) in the MAP4 (Glu)-treated group were stronger than those in the control and MAP4 (Ala)-treated groups (Fig. 4M and O, *p* < 0.01). These data demonstrated that the NLRP3 inflammasome in endothelial cells was markedly activated by p-MAP4 in vitro.

### p-MAP4 activated pyroptosis in vitro

**Fig. 5:**
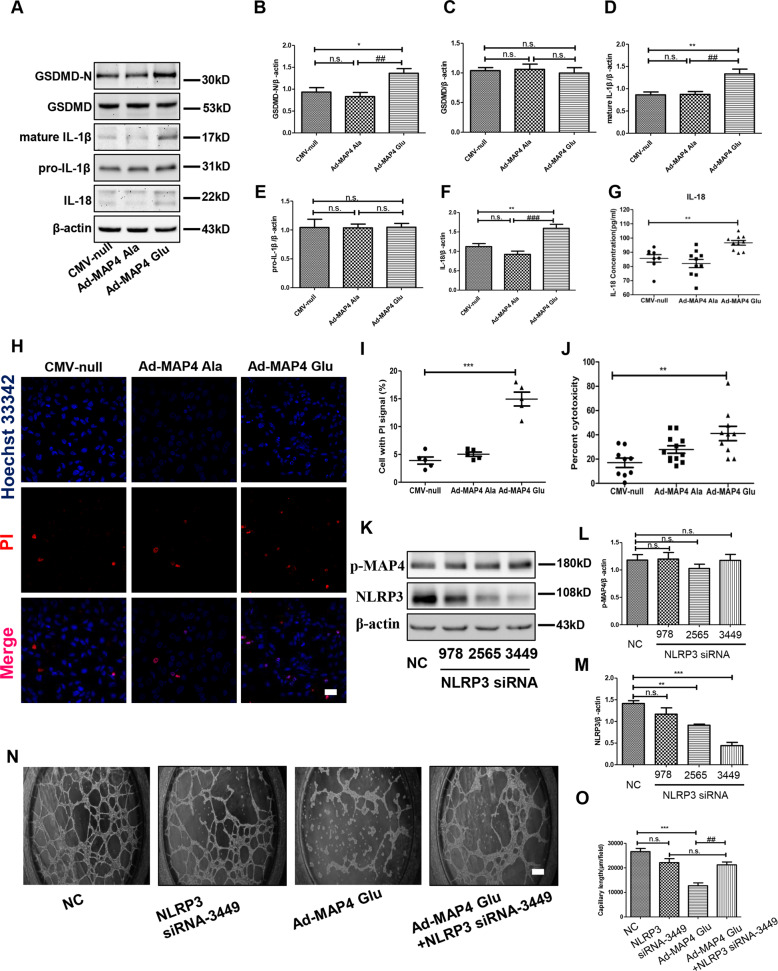
Pyroptosis is activated by p-MAP4 and NLRP3 suppression alleviated p-MAP4-induced angiogenesis inhibition in vitro. **A** Representative immunoblotting bands of GSDMD-N, GSDMD, pro-IL-1β, mature IL-1β, and IL-18 in HUVECs; *n* = 5. **B**–**F** The statistical analysis of GSDMD-N, GSDMD, pro-IL-1β, mature IL-1β, and IL-18. *p < 0.05 and **p < 0.01 versus the CMV-null group. ^##^*p* < 0.01 and ^###^*p* < 0.001 versus the Ad-MAP4 (Ala) group. N.S. represents no statistical difference; *n* = 5. **G** A scatter diagram showing the concentration of IL-18 in the medium of HUVECs. **p < 0.01 versus the Con group; *n* = 8. **H** Representative confocal images of co-staining with Hoechst 33342 and PI in HUVECs. Scale bar, 10 µm; *n* = 3. **I** A scatter diagram showing the statistical analysis of HUVECs with PI signal (%). ***p < 0.001 versus the CMV-null group; *n* = 3. **J** A scatter diagram showing cytotoxicity, represented by the release of LDH, in HUVECs. **p < 0.01 versus the Con group; *n* = 8. **K** Representative immunoblotting bands of NLRP3 and p-MAP4 after NLRP3 siRNA treatment; *n* = 5. **L** The statistical analysis of p-MAP4 in HUVECs treated by NLRP3 siRNAs. N.S. represents no statistical difference; *n* = 5. **M** The statistical analysis of NLRP3 in HUVECs treated by NLRP3 siRNAs. N.S. represents no statistical difference; **p < 0.01 versus the NC group; ***p < 0.001 versus the NC group; *n* = 5. **N** The representative image of tube formation assay in HUVECs treated with Ad-MAP4 (Glu) with or without NLRP3 siRNA. Scale bar, 10 µm; *n* = 3. **O** The statistical analysis of capillary length. **p < 0.01 and ***p < 0.001 versus the CMV-null group. ^##^*p* < 0.01 versus the Ad-MAP4 (Glu) + siNLRP3 group. N.S. represents no statistical difference; *n* = 3.

As NLRP3 inflammasome was activated by p-MAP4 in vitro, we then verified the role of p-MAP4 in pyroptosis in vitro. Therefore, we detected pyroptosis-related proteins using different measures. As shown in Fig. 5A, the expression of GSDMD, GSDMD-N, pro-IL-1β, mature IL-1β, and IL-18 were assayed through western blotting. As shown in Fig. 5A–F, we observed the increased levels of GSDMD-N, mature IL-1β, and IL-18 following treatment with the MAP4 (Glu) adenovirus compared with the control group (*p* < 0.05); no visible variation was noted in MAP4 (Ala)-treated group. Furthermore, we used a specific ELISA kit to measure the concentration of mature IL-18 in the medium. Accordingly, we found an increased concentration of IL-18 in the group treated with Ad-MAP4 (Glu) compared with the control group (Fig. 5G, *p* < 0.01). No marked variation was observed in the MAP4 (Ala) adenovirus-treated cells (Fig. 5B). As one essential criterion of pyroptosis, we employed co-staining with PI and Hoechst 33342. Our results showed that the percentage of PI-positive cells was significantly increased following treatment with the MAP4 (Glu) adenovirus, whereas the MAP4 (Ala) adenovirus had no evident effect (Fig. 5H and I, *p* < 0.01). The cytotoxicity, represented by the release of LDH, was shown to be amplified by Ad-MAP4 (Glu) compared with the control group (Fig. 5J), whereas no statistically significant variation was induced following treatment with the MAP4 (Ala) adenovirus (Fig. 5J). These data confirmed that p-MAP4 could trigger pyroptosis in endothelial cells.

### Suppressing NLRP3-related pyroptosis alleviated p-MAP4-induced angiogenesis inhibition

Next, to further verify the effects of NLRP3-related pyroptosis on p-MAP4 inhibited angiogenesis, we utilized NLRP3 siRNA to silence NLRP3 and its related pyroptosis. As shown in Fig. 5K, NLRP3 siRNA significantly reduced NLRP3 expression. Furthermore, a tube formation assay was conducted, and the results indicated that NLRP3 siRNA could significantly alleviate p-MAP4-induced angiogenesis inhibition (Fig. 5L). Moreover, capillary length analysis showed that NLRP3 siRNA prolonged the shortened capillary length induced by p-MAP4 (Fig. 5M, *p* < 0.01). Thus, the results suggest that p-MAP4 inhibited angiogenesis through activating NLRP3-related pyroptosis.

## Discussion

**Fig. 6:**
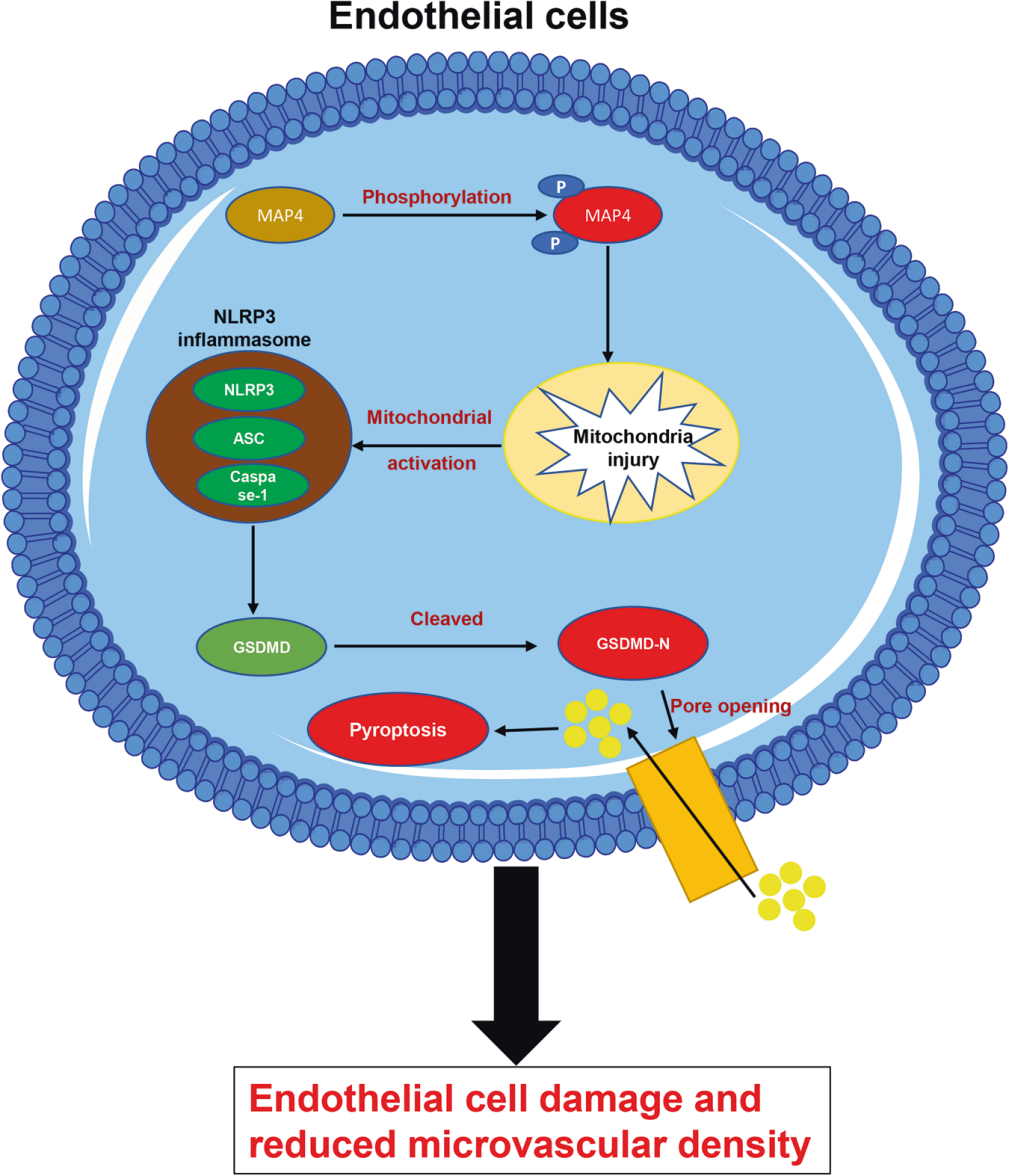
Schematic illustrating the reduction of cardiac microvascular intensity by the phosphorylation of MAP4. The phosphorylated MAP4 activates the NLRP3 inflammasome, further triggering pyroptosis in endothelial cells. This results in angiogenic disorder and reduced cardiac microvascular intensity.

The phosphorylation of MAP4 (p-MAP4) causes cardiac remodeling in an age-dependent manner, in which the cardiac microvascular endothelium is a vital mediator. The cardiac microvascular density acts as a critical criterion of the biological behaviors of the cardiac microvascular endothelium. Therefore, we analyzed the role of the phosphorylation of MAP4 in cardiac microvascular density and investigated the underlying mechanisms. Our findings revealed that p-MAP4 impaired cardiac endothelial cells and reduced cardiac microvascular density. Moreover, our data also demonstrated that this phenomenon was mainly attributed to NLRP3-related pyroptosis (Fig. 6). Thus, MAP4 might play a major role in maintaining microvascular density and simultaneously serve as a target of a new potential therapeutic strategy for cardiac remodeling.

Our previous research indicated that phosphorylation of MAP4 (S737 and S760 in mice) induced cardiac remodeling in an age-dependent manner, with endothelial cell dysfunction playing an important role in pathological cardiac remodeling [1, 19]. Therefore, we explored the variation in microvascular density, one major biological behavior of endothelial cells, in the myocardium of MAP4 KI mice. In this study, we first discovered that VEGF/VEGFR2 and ANG2/TIE2, two principal angiogenic signaling pathways, were significantly inhibited by the phosphorylation of MAP4, both in young and aged mice. Consistent with this, and as a result of inhibited angiogenesis, we noted that cardiac microvascular intensity was reduced, both in young and aged MAP4 KI mice, thus offering a rational explanation for p-MAP4-induced cardiac remodeling [1]. Based on our results, NLRP3-related pyroptosis might serve as an explanation for its activation in the myocardium of young and aged MAP4 KI mice. Previously published studies have identified the regulation of inflammasome-related pyroptosis in microvascular-related diseases, including lung destruction following traumatic brain injury, diabetic retinopathy, and the disruption of the blood-brain barrier [23–25]. Nevertheless, more direct but contradictory evidence showed that phosphorylation of MAP4 could promote the proliferation and migration of endothelial cells under hypoxia [5]. Therefore, it is urgent to address the multiple roles of the phosphorylation of MAP4 on endothelial cells and determine whether it reduces cardiac microvascular density via NLRP3-related pyroptosis.

The NLRP3 intracellular sensing protein is known to form a macromolecular structure called the NLRP3 inflammasome, which stimulates the innate immune response. The NLRP3 inflammasome is a complex consisting of NLRP3, ASC, and caspase-1. Excessive activation of the NLRP3 inflammasome and subsequent release of IL-1β have been shown to play a major role in the occurrence, development, and complications of atherosclerosis, acute myocardial infarction, non-ischemic injury to the myocardium, and heart failure [20]. To date, an increasing number of studies have identified that microtubule dynamics are closely associated with NLRP3 inflammasome activation. For example, the assembly of NLRP3 and its adapter ASC then requires the accumulation of acetylated α-tubulin on the microtubules to form optimal sites near the endoplasmic reticulum [26, 27]. Thus, microtubule depolymerization attenuates the activation of the NLRP3 inflammasome. Inversely, our in vitro experiment demonstrated that phosphorylation of MAP4 activated the NLRP3 inflammasome, manifested as the augmented expression of NLRP3, ASC, and cleaved caspase-1 (p10). In light of the phosphorylation of MAP4 being confirmed as an effective contributor to microtubule depolymerization, we could not explain this result from the aspect of microtubule depolymerization [6]. Therefore, a reasonable explanation for this should focus on p-MAP4 itself, and more specifically, on the subsequent effects of p-MAP4 after it detaches from microtubules at the principal entry point. Among these effects, the mitochondrial translocation and impairment have been well verified by our group [11, 22]. We found that p-MAP4-induced mitochondrial impairment was also evident in cardiac endothelial cells. Mitochondria, the metabolic powerhouses, are known to be caches for danger-associated molecular patterns and pathogen-associated molecular pattern-like molecules that elicit potential innate immune responses [28]. Thus, continuous mitochondrial injury caused by these molecules would initiate inappropriate or sustained inflammasome signaling, named mitochondrial activation of the inflammasome [28]. In detail, p-MAP4 made the outer membrane vesicles to open, leading to mitochondrial apoptosis, mitochondrial DNA release, and potassium ion efflux [22, 29, 30]. Therefore, mitochondrial impairment induced by the phosphorylation of MAP4 should be regarded as the core mechanism of the p-MAP4-induced activation of the NLRP3 inflammasome in HUVECs.

As an inflammation-related programmed cell death process, pyroptosis is a morphologically and mechanistically unique form of programmed cell death compared with others, such as apoptosis and autophagic cell death. Pyroptosis is primarily driven by caspase-1, which is the principal component of the NLRP3 inflammasome, and this kind of pyroptosis is named NLRP3-related pyroptosis [21]. After the transformation of caspase-1 into its active form, the cleaved caspase-1 further cleaved the GSDMD into GSDMD-N. Once GSDMD-N is translocated to the inner membrane and oligomerization occurs, the membrane pore is generated, followed by cell lysis and the secretion of proinflammatory intracellular contents, including IL-18 and IL-1β [21]. Our data showed that the phosphorylation of MAP4 activated pyroptosis manifested as increased GSDMD-N, IL-1β, IL-18, and LDH release in HUVECs. Based on the phosphorylation of MAP4 triggering the activation of the NLRP3 inflammasome, this result is considered rational because pyroptosis is the downstream process of the NLRP3 inflammasome. Nevertheless, we attempted to determine the association between MAP4 and pyroptosis. More specifically, the levels of phosphorylation of MAP4 and GSDMD-N were shown to positively correlate. Regarding this correlation, we observed that the explanation lay behind a specific domain that is phosphorylated. In the current study, the phosphorylation sites, which belong to the microtubule-binding domain (MTBD) of MAP4, were S737 and S760 in mice (corresponding to S768 and 787 in humans) [6]. After phosphorylation at its MTBD domain, MAP4 has been reported to become detached from MT, potentially simultaneously increasing the function of the projection (PJ) domain. The PJ domain has been predicted to possess several unexploited functions [31]. Whether the motif interacting with GSDMD-N is contained in the PJ domain and whether phosphorylation at MTBD functions as an inducer of the function of the PJ domain remains to be clarified. Based on our data, the NLRP3 inflammasome was identified to contribute to pyroptosis activation driven by the phosphorylation of MAP4. However, the major limitation of the research is that the MAP4 KI mouse model is systemic. Our previous research verified that no statistical differences exist between the biochemical parameters of two genotypes of mice [1]. However, we could not completely exclude the potential influences of other cells, tissues, and organs on microvascular density. The time- and endothelial-specific MAP4 phosphorylation mice are required to address this issue.

In conclusion, phosphorylation of MAP4 reduced cardiac microvascular density by triggering NLRP3-mediated pyroptosis. This induction of pyroptosis is attributed to the activation of the NLRP3 inflammasome. Furthermore, GSDMD-N, the main effector molecule of pyroptosis, was shown to form pores on the membrane of endothelial cells, resulting in cell death, inhibition of angiogenesis, and reduction of cardiac microvascular intensity. These data provide another possible mechanism for MAP4 phosphorylation-induced age-dependent cardiac remodeling, further expanding our knowledge on the effects of the phosphorylation of MAP4 in cellular processes.

## Materials and methods

### Ethics statement

All animal experiments were implemented in accordance with the Guide for the Care and Use of Laboratory Animals announced by the US National Institutes of Health (NIH Publication, 8th Edition, 2011) and were granted permission by the Animal Experiment Ethics Committee of the Army Medical University (the Third Military Medical University). The ethics approval number is AMUWEC20201489.

### Animal studies

Healthy young male C57BL6 mice (8–10-wk old, weighing 18–22 g) and aged C57BL6 mice (~70-wk old, 28–33 g) were purchased from the Animal Center, Army Medical University (Third Military Medical University). We had previously generated MAP4 KI mice in our lab [1]. Likewise, MAP4 KI mice contained both 8–10-wk old and ~70-wk old animals, with a corresponding weight of 18–22 g and 28–33 g, respectively. All animals were fed a standard chow, watered, and housed under a 12 h light/dark cycle. All animals were allowed to adapt for 1 wk before the experiments and were randomly divided into four groups: Con-Young, MAP4 KI-Young, Con-aged, and MAP4 KI-aged. Mice were euthanized to collect the myocardium samples (10 mg) for transmission electron microscopy (TEM) or immunoblot analysis. The investigators were blinded to the group allocation.

### Cell culture

Human umbilical vein endothelial cells (HUVECs) were obtained from the American Type Culture Collection (ATCC, USA) and cultured in RPMI 1640 medium (SH30809, HyClone) containing 10% fetal bovine serum (FBS; 10100139, Gibco), 100 U/mL penicillin, and 100 μg/mL streptomycin (Beyotime, China). Cells were incubated at 37 °C in a 5% CO_2_ atmosphere with 95% humidity.

### Construction and transduction of recombinant adenovirus

The MAP4 (Glu) (S768/787E) and MAP4 (Ala) (S768/787A) adenoviruses were described previously [6, 22]. HUVECs cultured in 6-, 24-, or 96-well plates were transfected with these adenoviruses for 48 h, with CMV-null adenoviruses being used as negative controls. Infection efficiency was determined by western blotting.

### Western blotting assay

Left ventricular (LV) myocardium samples or HUVECs were harvested in RIPA buffer supplemented with protease inhibitor tablets (Beyotime) and then sonicated on ice. The lysate was then centrifuged at 14,000 rpm for 15 min at 4 °C, and the supernatant was collected. Protein concentrations were assayed according to a previously published protocol using Quick Start™ Bradford 1x dye reagent (Bio-Rad, USA) [5]. Proteins were separated using an SDS-PAGE gel (Bio-Rad) and then transferred to PVDF membranes (Millipore, USA), where they were blocked with 5% skim milk. Then, membranes were incubated at 4 °C overnight with the corresponding primary antibodies and HRP-conjugated secondary antibodies. Specific protein bands were visualized using the Western Bright Sirius chemiluminescent HRP substrate (Pierce, USA) with a ChemiDoc XRS image detector (Bio-Rad). The following antibodies were used in this experiment: anti-NLRP3 (Invitrogen, 1:1000), anti-ASC (Santa Cruz, 1:100), anti-caspase-1 (Proteintech, 1:100), anti-GSDMD-N (CST, 1:1000), anti-IL-1β (Abcam, 1:1000), anti-IL-18 (Abcam, 1:1000), anti-β-actin (Proteintech, 1:5000), anti-phospho-MAP4 (p-MAP4) (GL Biochem, 1:1000), anti-p-MAP4 (S787) (GL Biochem, 1:1000), anti-p-MAP4 (S768) (Biolegend, 1:1000), and anti-MAP4 (Affinity, 1:1000).

### Electron microscopy

As previously described [1], the LV myocardium was fixed in 2.5% glutaraldehyde, dehydrated, sliced with a vibratome, recut on a microtome, and stained with uranyl acetate and lead citrate overnight. Sections of cardiomyocytes were visualized by TEM (TECNAI 12, Philips, Amsterdam, Netherlands).

### Immunofluorescence and confocal microscopy

HUVECs were plated on glass coverslips, fixed with 4% paraformaldehyde for 30 min, and blocked with 5% bovine serum albumin (BSA) in PBS for 1 h at 25 °C. Then, cells were initially incubated with specific primary antibodies at 4 °C overnight and subsequently incubated with the corresponding secondary antibodies for 1 h at 37 °C. Nuclei were stained for 5 min with DAPI (#40011, Biotium, Inc) or Hoechst 33342 (#H1399, Invitrogen) or PI (propidium iodide, #P1304MP, Invitrogen). Cells were imaged using a confocal microscope. The following primary antibodies were used in this experiment: mouse monoclonal anti-NLRP3 (Invitrogen, 1:1000), anti-ASC (Santa Cruz, 1:100), and anti-caspase-1 (Proteintech, 1:100). The Alexa Flour-488 donkey anti-rabbit (A21206) secondary antibody was purchased from Invitrogen. After that, Pearson R value and fluorescence intensity were analyzed using Image J software.

### Cell toxicity assays

Cytotoxicity was detected using a CytoTox-ONE™ C homogeneous membrane integrity assay kit (Promega, USA), a fluorometric method used to measure the amount of lactate dehydrogenase (LDH) released into the medium from inactive cells. All experiments were performed according to the manufacturer’s instructions. The percentage of LDH released into the medium was used to evaluate cytotoxicity.

### ELISA

Specific ELISA kits were purchased from the Proteintech Group to detect the concentrations of interleukin-18 (IL-18) and vascular endothelial growth factor (VEGF) in various media. Detection procedures were conducted according to the manufacturer’s instructions.

### Tube formation assay

Before the experiment, Matrigel (#356231, Corning, USA) was paved on µ-slide angiogenesis ibiTreat (#81506, IBIDI). Next, after transfected with adenovirus (CMV-null, Ad-MAP4 Ala, and Ad-MAP4 Glu) for 48 h or siRNA (NC and siNLRP3) for 24 h, the HUVECs were collected. 1 × 10^4^ HUVECs were seeded on Matrigel surface and cultured for 8 h. The tube formation was then observed and photographed using a microscope. Then, the total length of tubes was calculated using Image J (Fiji) software.

### Gene silencing with siRNAs

NLRP3 siRNA was purchased from Genepharma (Shanghai, China). HUVECs were transfected with the target siRNAs or the negative control siRNAs with Lipofectamine 2000 (Invitrogen, USA), referring to the manufacturer’s instruction. All experiments were performed after transfection for 24 h.

**Table:** 

	Sequence (5′–3′)	Sequence (3′–5′)
NLRP3-homo-978	CAACAGGAGAGACCUUUAUTT	AUAAAGGUCUCUCCUGUUGTT
NLRP3-homo-2565	GCUGCUGAAAUGGAUUGAATT	UUCAAUCCAUUUCAGCAGCTT
NLRP3-homo-3449	GUGCGUUAGAAACACUUCATT	UGAAGUGUUUCUAACGCACTT

### Statistical analysis

The SPSS software was used for statistical analysis. Data represent mean ± SEM. Significant differences were analyzed using the unpaired Student’s *t*-test, one-way or two-way analysis of variance (ANOVA) followed by post hoc tests. Scatter diagrams were created using GraphPad. A *p* < 0.05 was considered statistically significant for all comparisons.
